# Allelic mapping bias in RNA-sequencing is not a major confounder in eQTL studies

**DOI:** 10.1186/s13059-014-0467-2

**Published:** 2014-09-20

**Authors:** Nikolaos I Panousis, Maria Gutierrez-Arcelus, Emmanouil T Dermitzakis, Tuuli Lappalainen

**Affiliations:** Department of Genetic Medicine and Development, University of Geneva Medical School, Geneva, Switzerland; Institute of Genetics and Genomics in Geneva (iGE3), University of Geneva Medical School, Geneva, Switzerland; Swiss Institute of Bioinformatics, Geneva, Switzerland; Center of Excellence for Genomic Medicine Research, King Abdulaziz University, Jeddah, Saudi Arabia; Department of Genetics, Stanford University School of Medicine, Stanford, USA; New York Genome Center, New York, NY USA; Department of Systems Biology, Columbia University, New York, NY USA

## Abstract

**Background:**

RNA sequencing (RNA-seq) is the current gold-standard method to quantify gene expression for expression quantitative trait locus (eQTL) studies. However, a potential caveat in these studies is that RNA-seq reads carrying the non-reference allele of variant loci can have lower probability to map correctly to the reference genome, which could bias gene quantifications and cause false positive eQTL associations. In this study, we analyze the effect of this allelic mapping bias in eQTL discovery.

**Results:**

We simulate RNA-seq read mapping over 9.5 M common SNPs and indels, with 15.6% of variants showing biased mapping rate for reference versus non-reference reads. However, removing potentially biased RNA-seq reads from an eQTL dataset of 185 individuals has a very small effect on gene and exon quantifications and eQTL discovery. We detect only a handful of likely false positive eQTLs, and overall eQTL SNPs show no significant enrichment for high mapping bias.

**Conclusion:**

Our results suggest that RNA-seq quantifications are generally robust against allelic mapping bias, and that this does not have a severe effect on eQTL discovery. Nevertheless, we provide our catalog of putatively biased loci to allow better controlling for mapping bias to obtain more accurate results in future RNA-seq studies.

**Electronic supplementary material:**

The online version of this article (doi:10.1186/s13059-014-0467-2) contains supplementary material, which is available to authorized users.

## Background

Analysis of gene expression variation and its genetic causes is essential for better understanding of phenotypic variation and susceptibility to complex traits and diseases [1,2]. One of the most popular methods to find genetic variants that affect gene expression levels is expression quantitative trait locus (eQTL) analysis [3–5] that is more and more often based on RNA sequencing (RNA-seq) [6,7], which has become the gold-standard method to quantify gene expression. However, gene expression quantification from RNA-seq can potentially be biased by genetic variation affecting the mapping of RNA-seq reads, as those reads that carry the non-reference allele can have a lower probability of mapping correctly to the reference genome [8]. This is analogous to single-nucleotide polymorphisms (SNPs) in probes of expression microarrays, which is a relatively well-documented technical problem [9,10]. Similar problems may arise in alignment of ChIP-Seq reads and chromatin state QTL mapping. In RNA-seq analysis, accounting for genotype-dependent mapping bias has been important for obtaining more accurate and reliable results from analysis of allelic specific expression (ASE) [8,11–14], allelic specific binding (ASB) [12], and DNaseI sensitivity QTLs [15]; nevertheless, similar analyses have not been done for eQTLs.

In this study we examined if allelic mapping bias of RNA-seq reads is a confounding factor in eQTL analysis. We estimated mapping bias in SNPs and insertions-deletions (indels) from Europeans (CEU, GBR, TSI, FIN, IBS) of 1000 Genomes [16] Phase 1 data by simulations. We then assessed the effects of this mapping bias on exon quantification and subsequently on eQTL discovery using an RNA-seq dataset. Our results suggest that mapping bias does not severely affect eQTL findings and gene expression quantification. However, a small proportion of eQTL associations are likely to be false positives due to allelic mapping bias, and correcting for these effects will lead to more accurate results.

## Results

### Different alignment strategies to identify mapping bias

**Figure 1:**
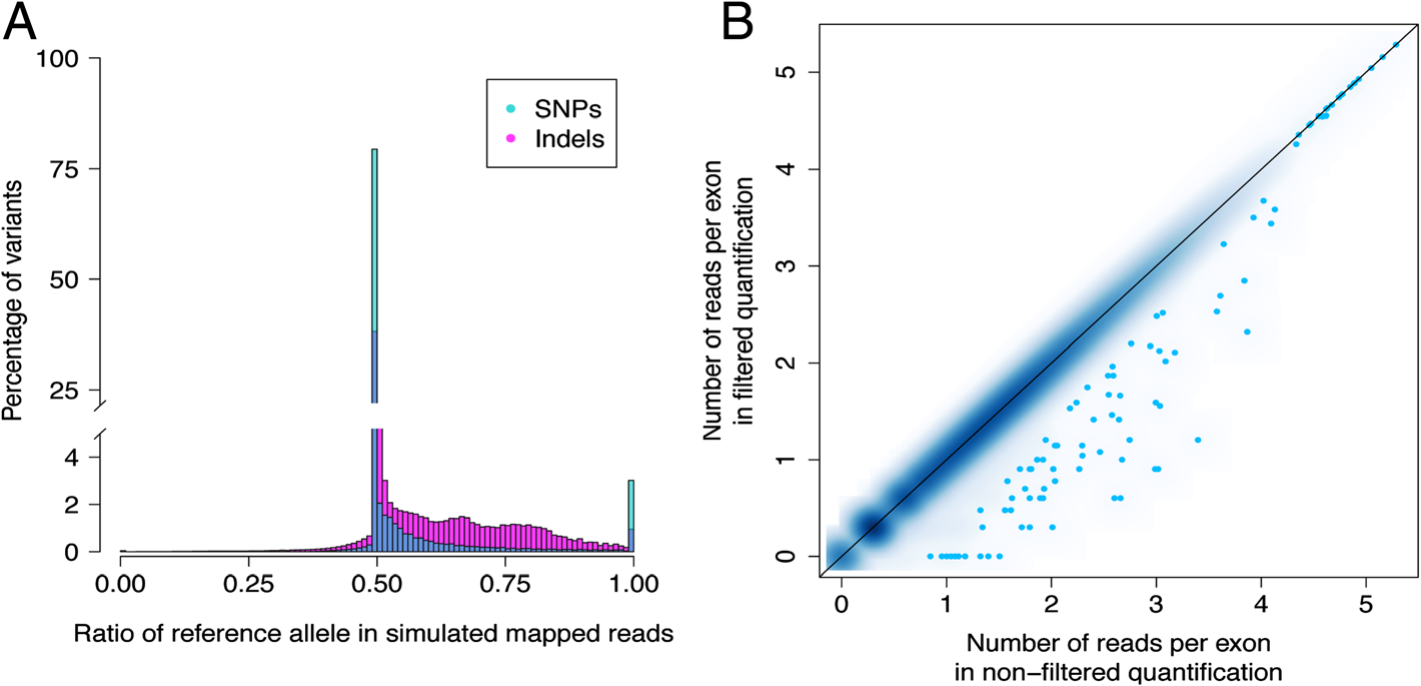
**Estimation of mapping bias and its effect on expression quantifications. (A)** Mapping bias in SNPs and indels of 1000 Genomes estimated by simulated single-end 50 bp RNA-seq reads based on the genome sequence and aligned with BWA. **(B)** The impact of filtering for reads with simulated mapping bias on exon quantifications (log10 scale).

**Figure 2:**
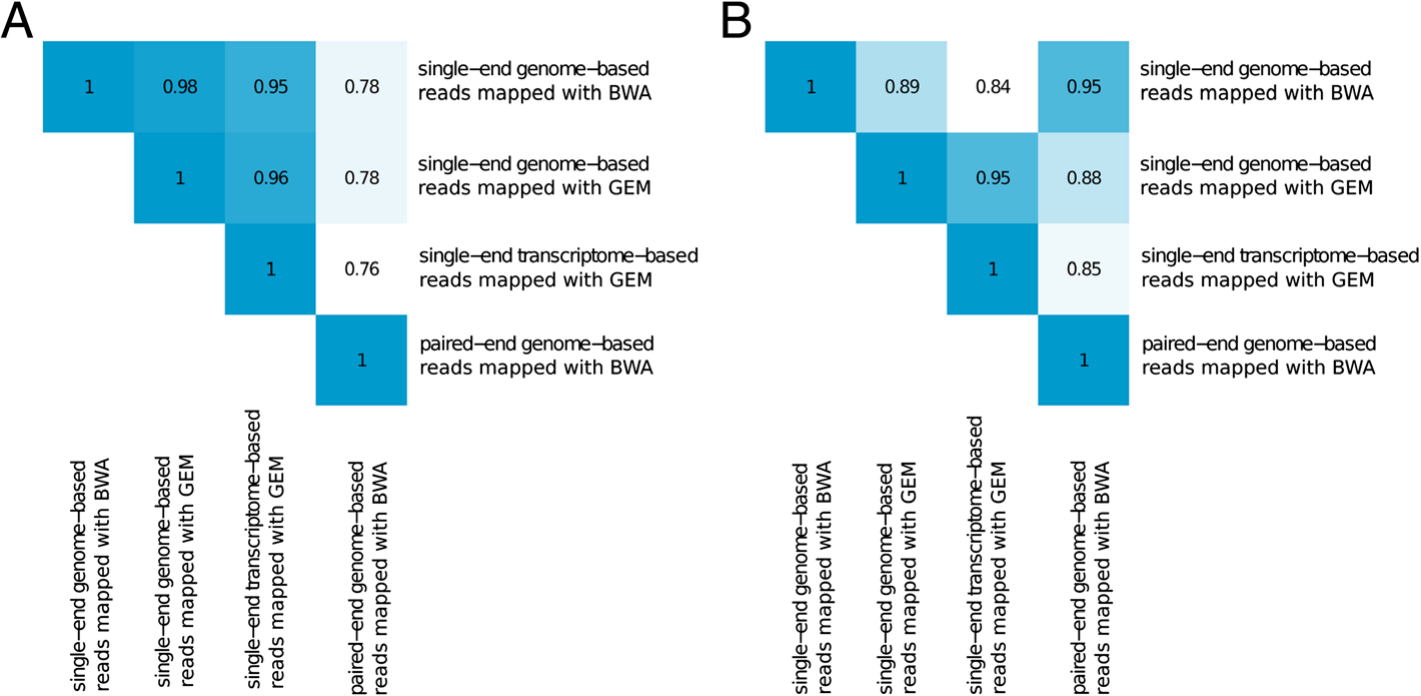
**Correlation of mapping bias with different alignment methods and read types. (A, B)** Spearman correlation of reference allele ratio for SNPs **(A)** and indels **(B)** in simulations with different mappers and different read building strategies.

For 9.5 M variant sites, we first created simulated single-end 50 bp RNA-seq reads, first using the genome sequence as is for a total of 456,210,044 unique start sites, with reads carrying both reference and non-reference haplotypes. After alignment of these reads to the reference genome with BWA [17], we found that as much as 13.86% (63.2 M) of the unique start sites showed unequal mapping of reads carrying different alleles with 78.30% coming from SNPs and 21.70% coming from indels. Summarizing this at the level of variants, 12.6% (1,088,730) of the SNPs and 45.6% (397,399) of the indels had >5% difference in the mapping rate of all overlapping reference and non-reference reads (Figure 1A, Table 1), with the bias being significantly higher in indels (Mann-Whitney *P* value <2.2e-16; Figure 1A). However, because indels are much less common, the majority of variants causing mapping bias are actually SNPs. In 96.1% of the biased variants, the bias favored the reference allele, which is the only possible direction for those without any flanking variants within the 50 bp range. However, if the reference allele of one variant is linked to an alternative allele of a second, flanking variant, it is possible to have mapping bias in favor of the non-reference allele of the first variant due to secondary effect, which we observe in a small proportion of cases (3.89%). We provide the simulation results as a resource for future studies, both summarized per variant and as a list of biased start sites (Additional file 1). To verify the robustness of these results to the choice of the mapper, we performed the alignment also with the GEM mapper [18] by using the GEMTools pipeline [19], and obtained similar results (Table 1, Additional file 1: Table S1). Specifically, the number of unique start sites that showed unequal mapping of reads carrying different alleles was 67.1 M with GEM, of which 61.1 M were shared with the biased start sites identified by BWA. Moreover, for a given variant we compared the ratio of reference allele in simulated reads mapped with BWA and GEM. The ratios were highly correlated (rho =0.98 for SNPs and 0.88 for indels, Figure 2A and B) suggesting that the choice of mapper has only minor effects.

**Table 1:** **Simulated bias in variant sites that are polymorphic with MAF >1% in Europeans, from single-end read simulations based on genome sequence and aligned with BWA**

	**SNPs**	**Indels**	**Total**
	**(90.84% of total)**	**(9.16% of total)**	
**Variants (n)**	8,650,740	872,262	9,523,002
**Variants with >0% bias**	1,822,445	550,423	2,372,868
	(21.06%)	(63.10%)	(24.91%)
**Variants with >5% bias**	1,088,730	397,399	1,486,129
	(12.58% of SNPs)	(45.56% of indels)	(15.60% of total)

**Figure 3:**
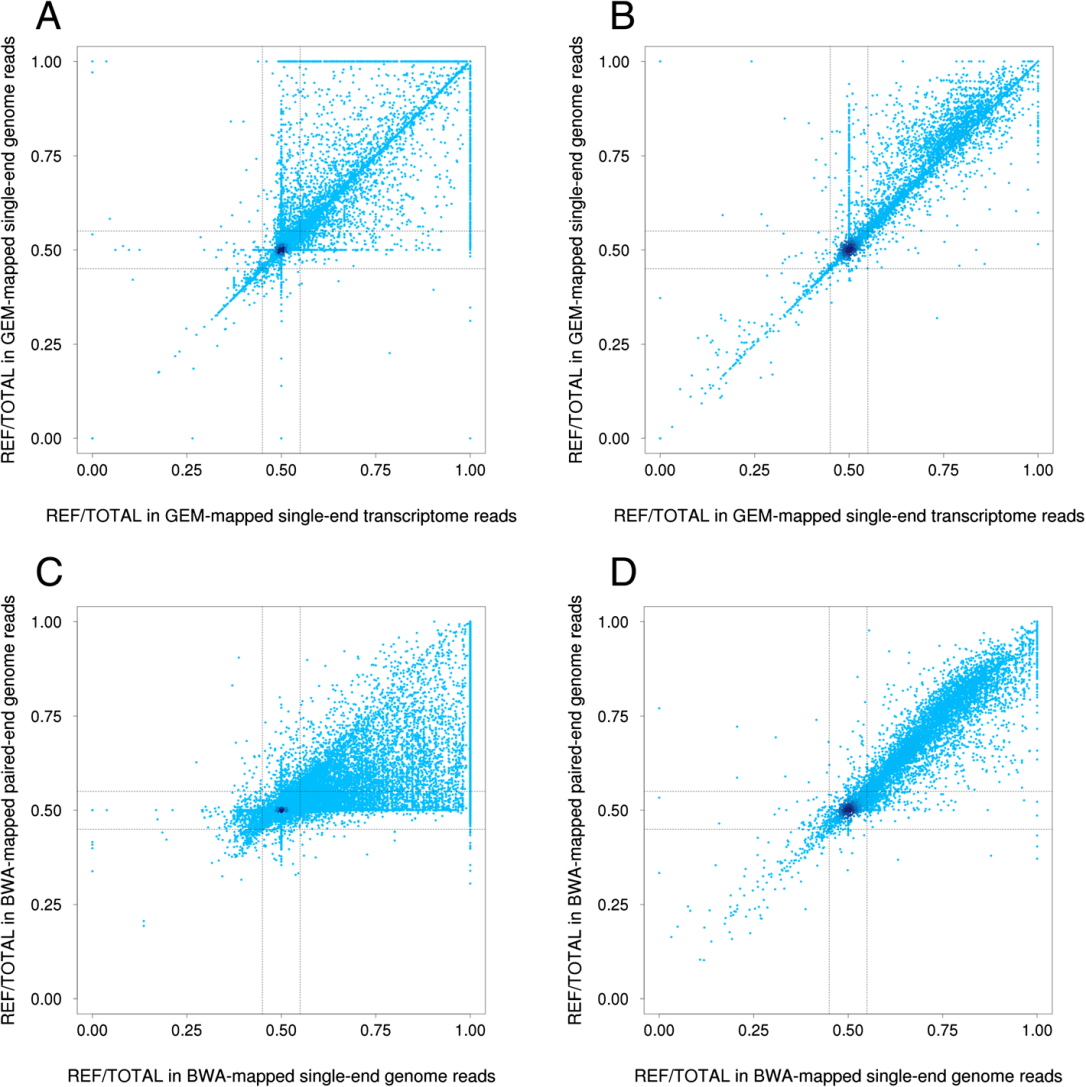
**Comparison of allelic ratios with different read types.** Reference allele ratio obtained from simulated reads over SNPs **(A, C)** and indels **(B, D)** comparing genome versus transcriptome-based reads **(A, B)** in single-end 50 bp reads mapped with the GEM mapper, and comparing single- versus paired-end reads **(C, D)** in genome-based 50 bp reads mapped with BWA. The dotted lines denote 5% difference of the ratio for reference/non-reference allele. The color scale from dark blue to light blue denote the density of the points.

The results above were based on reads simulated using the genome sequence, but a key property of RNA-seq are reads that span exon junctions. To assess if such split reads affect allelic mapping bias, we selected the most common transcript across tissues (see Methods) and simulated single-end 50 bp reads for variants in these transcripts using the annotated exon structure, and mapped the simulated reads with GEM that can map reads across splice junctions. We compared these results from variants in simulated transcripts to the genome-based simulations mapped with GEM, rather than BWA used in most analyses, in order to avoid confounding differences between mappers. The percentage of variants that show a >5% difference in the reference/non-reference mapping rate is similar for transcript- and genome-based simulations (Additional file 1: Table S2). The allelic ratios were highly correlated (rho =0.96 for SNPs and 0.95 for indels; Figures 2A and B, and 3A and B) and 97.00% of SNPs and 98.60% of indels that are biased in transcriptome-based simulations were also biased in genome-based data. These observations suggest that transcript structure and split reads in RNA-seq have a relatively minor effect on allelic mapping bias, and that even genome sequence-based estimates of biased loci are generally valid for RNA-seq.

Next, we tested the effect of single-end versus paired-end reads - the latter being the standard in modern RNA-seq. We simulated paired-end 50 bp reads with a fixed insert size for all coding variants that overlap the most common transcript across tissues as before, mapped the reads with BWA, and compared the results with the corresponding single-end results (Additional file 1: Table S2). As expected, we found fewer biased SNPs (5.15% in paired-end vs. 8.45% in single-end) and indels (43.03% in paired-end vs. 44.83% in single-end). While the overall correlation of the allelic ratios (paired-end vs. single-end) is high (rho =0.78 for SNPs and 0.95 for indels; Figure 2A and B), paired-end reads clearly reduce mapping bias for SNPs, but much less so for indels (Figure 3C and D). However, the high proportion of biased variants in paired-end data that are also biased in single-end data (92.5% for SNPs and 97.30% of indels) indicates that while single-end simulations are sometimes overly conservative for paired-end data, they find nearly all of the variants that would be biased in paired-end data.

### Effect of mapping bias in gene quantification and eQTL mapping

In order to investigate if the allelic mapping bias drives biased quantification of expression levels of exons and subsequently false eQTL discoveries, we combined the information from simulations with RNA-seq data from lymphoblastoid cells lines of 185 individuals from the Gencord project [20,21] (see Methods). In the standard quantification of exon expression levels, we quantified 78,595 exons (>0 reads in >90% of individuals). In this study, to analyze the effect of mapping bias in exon quantification and eQTL discovery, we removed all the RNA-seq reads that are mapped to biased start sites as indicated by our simulations, from each individual regardless of the genotype. We chose to use results from the single-end 50 bp genome-based mapped with BWA simulations, since they have fewer assumptions of transcript structure or insert size than the other simulations, and especially compared to the paired-end results these represent the worst-case scenario. After filtering away an average of 750,414 reads (85.40% filtered because of SNPs and 14.60% because of indels) per individual with start sites matching the 63.2 M start sites that were biased in our simulations, we quantified 78,281 exons (Additional file 1: Table S3), with an average of 6,408 exons per individual with at least one read removed. The quantifications between non-filtered and filtered data were extremely highly correlated (mean rho =0.998; Figure 1B), with the genes with low coverage in RNA-seq quantification having a proportionally similar loss of coverage as high coverage genes (Additional file 1: Figure S1). These results suggest that filtering reads with potential mapping bias hardly affects the overall pattern of exon quantification and does not significantly reduce the resolution or coverage of RNA-seq data.

**Figure 4:**
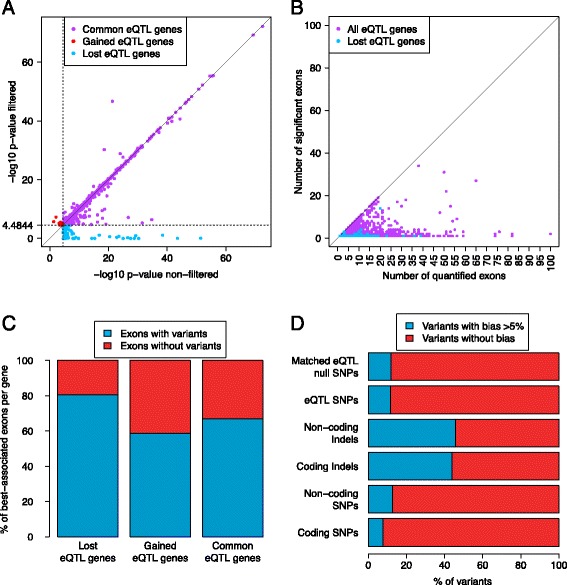
**Effect of mapping bias on eQTL discovery. (A)** Comparison of original and filtered *P* values (rho =0.92, *P* value <2.2e-16) shows that for the vast majority of the genes, the *P* values after filtering potentially biased reads are highly consistent with *P* values without filtering. Colors denote whether the gene with an eQTL in the original dataset was significant only before (lost) or only after (gained) filtering putatively biased reads or in both analyses (common). The dotted lines denote 10% FDR significance thresholds. **(B)** The number of exons per gene with significant associations as a function of the total number of quantified exons in the original, non-filtered dataset. **(C)** Proportion of the best-associated exon per gene with genetic variants (see also Additional file 1: Table S5). **(D)** Proportion of biased variants in six different categories based on the single-end genome based mapped with BWA simulated reads. Matched eQTL null is a random sample of variants matched to the distance from TSS of eQTLs.

Next, we examined if this change in quantifications due to removal of putative mapping bias affects eQTL discovery. In the standard, non-filtered dataset, we discovered 3,372 eQTL genes at 10% FDR. When using exon quantifications without reads in potentially biased start sites we observe 3,323 eQTL genes with the same *P* value threshold. These two eQTL sets are highly overlapping, both at the gene level, with 3,253 common genes (Table 2) and at the exon level, with 95.35% of non-filtered significant exons also called significant in the filtered set (Additional file 1: Table S4). In this paper, we call the eQTLs lost, gained, and common according to whether the gene with an eQTL in the original dataset was significant only before (lost) or only after (gained) filtering putatively biased reads or in both analyses (common). In 9.24% of lost eQTL genes or in 0.32% of all eQTL genes the log *P* value dropped by >20 after filtering biased reads, suggesting that these associations were clear false positives driven by allelic mapping bias (Figure 4A), and we list these genes (Additional file 1: Table S7). The *P* values before and after filtering were highly correlated (Figure 4A), but a sizeable fraction of genes had a log *P* value difference > =1 (49.57% of lost, 2.52% of common, and 14.28% of gained eQTL genes).

**Table 2:** **Number of eQTL genes detected before and after filtering for biased reads estimated from single-end read simulations based on genome sequence and aligned with BWA**

		**Non-filtered data**		
		**eQTL genes**	**Genes without eQTL**	**Total**
**Filtered data**	**eQTL genes**	3,253	70	3,323
	**Genes without eQTL**	119	8,800	8,919
	**Total**	3,372	8,870	12,242

To analyze further the differences in eQTL signals that were lost or gained when reads with putative mapping bias were removed, we first analyzed the number of significant exons per gene. We observed that most of the lost eQTL genes (92.4%) were associations to only a single significant exon (Figure 4B, Additional file 1: Figure S2). In contrast, eQTLs that were discovered only in filtered data, typically just above the *P* value threshold, had a high number of quantified exons (Additional file 1: Figure S3). Furthermore, we observed that exons in which the eQTL signal was lost by filtering were enriched for SNPs and indels within the exon compared to shared or gained exons (80.67% with variants in lost versus 58.57% in gained exons; Figure 4C; Additional file 1: Tables S5, S6; Fisher’s exact test *P* value =0.001). These results suggest that the cases where the eQTL association was lost by filtering are indeed enriched for false associations driven by mapping bias.

If an eQTL is driven by mapping bias, the biased variant itself (or strongly linked variants) is likely to show up as the most significant eQTL variant. If such false associations were widespread, eQTL variants would be expected to have higher simulated mapping bias. Thus, we investigated the simulated mapping bias of the most significant eQTL variant of all original eQTL genes, without filtering. Of the eQTL SNPs, 11.53% are biased (>5% difference in mapping of reference and non-reference reads), which is similar to the 11.87% of biased random SNPs that have been matched for distance from TSS (Mann-Whitney *P* value =0.2 for comparison of the full distributions of simulated bias, or Fisher’s exact test *P* value =0.6 for bias being >5% or <5%; Figure 4D). This supports the general observation suggesting that mapping bias is not a major driver of eQTL associations.

## Discussion

In this paper, we have addressed a potentially significant drawback of RNA-seq eQTL studies, with the reassuring result that eQTL detection is rarely affected by allelic mapping bias. While our simulations indicate that a substantial proportion of all genetic variants can give rise to mapping bias, such loci are less common in coding regions and thus the proportion of putatively biased RNA-seq reads is relatively low. The RNA-seq quantifications based on a much larger sequence target than probe sequences of arrays renders robustness against mapping bias, which is another important advantage over expression arrays [10].

However, the robustness of eQTL results to allelic mapping bias does not imply the same for all RNA-seq analyses. While the quantification of exons is robust against mapping biases, transcript quantifications might be affected, depending on the quantification method. Moreover, analysis of allelic effects is severely compromised by unequal mapping of reads due to the analyzed heterozygous variant itself or other flanking variants in the reads [8]. Our catalog of potentially biased variants can be used to remove suspicious sites from allelic expression [22] or binding analysis [23] as well. Furthermore, we have addressed only eQTLs for coding genes, but for example miRNA quantifications are based on shorter reads and repetitive targets. This makes these studies more vulnerable for mapping biases, necessitating more extensive approaches to account for mapping bias [22].

Our approach, with filtering potentially biased start sites and variants detected from the simulations, can be easily applied to other studies, and we provide the results of our simulations to enable this. These results are similar for different alignment methods, genome versus transcriptome models, and single- versus paired-end reads, suggesting that exact simulations according the precise RNA-seq assay used in each study is not always necessary. Our single-end based analyses also apply for ChIP-seq analyses [15,23] that usually rely on single-end reads. Our approach is intentionally stringent in using haplotypes of all variants with >1% frequency in equal proportions in the simulated read pool, regardless of their population frequency. However, not simulating all possible transcripts, not simulating sequencing errors, and errors in the indel calling of 1000 Genomes Phase 1 data may lead to some underestimation of the bias. Altogether, finding only a handful of eQTLs likely driven by mapping bias suggests that they are unlikely to be widespread. An alternative method to eliminate mapping biases is alignment to personalized references [12]. However, this is not only computationally challenging for large population-based RNA-seq studies but will take into account only genotyped variants, which is almost always far from a comprehensive representation of all variants, especially indels. Thus, a filtering strategy based on all known common variants, as in this study, is likely to be more comprehensive.

## Conclusions

Our results indicate that allelic mapping bias is not a major confounder in gene and exon quantifications based on RNA sequencing data, nor a major source of false positive eQTL findings. However, the possibility of false associations should be taken into account especially when analyzing individual loci. Furthermore, as sample sizes increase, even very slight biases can give rise to significant associations. Thus, estimating the effects of allelic mapping bias and accounting for that in analyses is one of the important steps towards efficient use of RNA-sequencing technology to measure the transcriptome and its variation.

## Methods

We first estimated allelic read-mapping bias by simulating RNA-seq reads with and without variant alleles based on the genome sequence. We analyzed 8,650,740 SNP and 872,262 indel variants from the European samples (CEU, GBR, TSI, FIN, IBS) in 1000 Genomes [16] Phase 1 data with minor allele frequency >1%. We created the potential 50 bp single-end reads overlapping these variants in all the observed haplotype combinations, yielding a total of 1,204,281,163 reads. We did not simulate sequencing errors due to the extremely large number of reads that would result from this. For simulating reads derived from the transcriptome rather than genome, we selected the most common transcript for each gene from the pilot phase dataset of the GTEx project [24] across all the tissues. Next, for all of these transcripts we identified all the variants that fully overlap its exons and we created all the possible 50 bp reads taking into account all the observed haplotype combinations and the annotated exon structure of the transcript. Additionally, we also simulated 50 bp paired-end reads with an insert size of 60 nucleotides (median insert size in Gencord Project [20,21]) based on the genome sequence, using the same subset of variants as in the transcript analysis.

The simulated reads were mapped to the hg19 reference genome [25] either with BWA [17] (single-end and paired-end genome-based reads) or with GEM [18] (single-end genome and transcriptome reads) and identified the read start positions where reads carrying different alleles (including reads with linked flanking variants) did not map equally.

To examine if quantification of expression levels of exons and eQTL discoveries are driven by mapping bias we incorporated the information obtained by our simulations with RNA-seq data from lymphoblastoid cells lines of 185 individuals from the Gencord project [20,21]. In the original analysis of this dataset, 49 bp paired-end reads (median 39 million per individual) were mapped to hg19 reference genome [25] with BWA [17]. Autosomal exons were quantified from raw read counts, and normalized by the total number of reads that mapped to exons and four technical covariates (for details, see Gutierrez-Arcelus *et al.* [21]) (Additional file 1: Table S3). These quantifications and genotype data from 5.6 M SNPs (Omni 2.5 M SNP array imputed to 1000 Genomes Phase 1) were used to map cis-eQTLs by Spearman rank correlation, with FDR adjusted to 10% by permutations. In this study, to analyze the effect of mapping bias in exon quantifications and eQTL discovery, we removed all the RNA-seq reads that are mapped to biased unique start sites as indicated by our simulations, from each individual regardless of the genotype. We then re-ran the analyses on the filtered data, using the same quantification, normalization, eQTL mapping procedure, and eQTL permutation threshold as before.

## Additional file

Additional file 1: Table S1. Simulated bias in variant sites that are polymorphic with MAF > 1% in Europeans by using single-end reads simulated based on the genome sequence and aligned with the GEM mapper. **Table S2.** Simulated bias in a subset of coding variant sites that are polymorphic with MAF > 1% in Europeans by using different read building strategies and aligners. **Table S3.** Effect of filtering on mapped reads and expression quantification. **Table S4.** Overlap of eQTL datasets (Non-filtered and filtered) in the exon level. **Table S5.** Numbers and percentages of variants within the best eQTL exons in the lost, gained and common eQTL genes. **Table S6.** Number of exons with and without variants in common, lost, and gained eQTL genes. **Table S7.** Genes that were highly significant before filtering biased reads with a difference in the -log10 of *P* value before and after filtering >20. **Figure S1.** Proportional loss of coverage in filtering biased reads for each gene, compared to the log10 of the number of reads per gene in the original data. **Figure S2.** Distribution of the number of exons that passed the *P* value threshold (significant exons) in eQTL genes in the non-filtered eQTL dataset for **(a)** lost eQTL genes, **(b)** gained eQTL genes, and **(c)** common eQTL genes. **Figure S3.** Distribution of the number of quantified exons in gained eQTL genes and all quantified genes (Mann-Whitney *P* value < 0.004). The higher the number of exons, the easier it is for a gene to pass the *P* value threshold and be characterized as eQTL gene. **Supplementary data.** The simulated data for all the alignment strategies used in this paper are freely and publicly available on our ftp server (ftp://jungle.unige.ch/Allelic_map_bias/).

## Abbreviations

ASB: Allele specific binding
ASE: Allele specific expression
DNAseI QTLs: Deoxyribonuclease I quantitative trait locus
eQTL: Expression quantitative trait locus
indels: Insertions and deletions
RNA-seq: RNA sequencing
SNPs: Single nucleotide polymorphisms
