# Supplementary material for: Allelic mapping bias in RNA-sequencing is not a major confounder in eQTL studies
Source: Genome Biol. 2014 Sep 20;15(9):467. doi: 10.1186/s13059-014-0467-2 (PMC4212091; doi:10.1186/s13059-014-0467-2)
Supplement: Additional file 1: Table S1. — Simulated bias in variant sites that are polymorphic with MAF > 1% in Europeans by using single-end reads simulated based on the genome sequence and aligned with the GEM mapper. Table S2. Simulated bias in a subset of coding variant sites that are polymorphic with MAF > 1% in Europeans by using different read building strategies and aligners. Table S3. Effect of filtering on mapped reads and expression quantification. Table S4. Overlap of eQTL datasets (Non-filtered and filtered) in the exon level. Table S5. Numbers and percentages of variants within the best eQTL exons in the lost, gained and common eQTL genes. Table S6. Number of exons with and without variants in common, lost, and gained eQTL genes. Table S7. Genes that were highly significant before filtering biased reads with a difference in the -log10 of P value before and after filtering >20. Figure S1. Proportional loss of coverage in filtering biased reads for each gene, compared to the log10 of the number of reads per gene in the original data. Figure S2. Distribution of the number of exons that passed the P value threshold (significant exons) in eQTL genes in the non-filtered eQTL dataset for (a) lost eQTL genes, (b) gained eQTL genes, and (c) common eQTL genes. Figure S3. Distribution of the number of quantified exons in gained eQTL genes and all quantified genes (Mann-Whitney P value < 0.004). The higher the number of exons, the easier it is for a gene to pass the P value threshold and be characterized as eQTL gene. Supplementary data. The simulated data for all the alignment strategies used in this paper are freely and publicly available on our ftp server (ftp://jungle.unige.ch/Allelic_map_bias/). [file 13059_2014_467_MOESM1_ESM.pdf]

Table S1. Simulated bias in variant sites that are polymorphic with MAF>1% in Europeans by using single-end reads simulated based on the genome sequence and aligned with the GEM mapper.

	SNPs (90.83% of total)	Indels (9.17% of total)	Total
**Number of variants**	8,651,677	873,357	9,525,034
**Variants with >0% bias**	19,772,19 (22.85%)	526,427 (60.27%)	2,503,646 (26.28%)
**Variants with >5% bias**	1,230,971 (14.22% of SNPs)	388,085 (44.43% of indels)	1,619,056 (16.99 % of total)

Numbers of variants sites (SNPs and indels) that show >0% or >5% difference in the mapping ratio of the reference and nonreference allele in simulations.

Table S2. Simulated bias in a subset of coding variant sites that are polymorphic with MAF>1% in Europeans by using different read building strategies and aligners.

	SNPs (93.13% of total)	Indels (6.87% of total)	Total
**Number of variants**	183,189	13,507	196,696
**Variants with >0% bias Single-end genome-based reads mapped with BWA**	25,267 (13.79%)	8,220 (60.85%)	33,487 (17.02%)
**Variants with >0% bias Single-end genome-based reads mapped with GEM**	27,708 (15.12%)	7,668 (56.77%)	35,376 (17.98%)
**Variants with >0% bias Single-end transcriptome-based reads mapped with GEM**	33,846 (18.48%)	7,589 (56.18%)	41,435 (21.06%)
**Variants with >0% bias Paired-end genome-based reads mapped with BWA**	23,921 (13.06%)	8317 (61.57%)	32238 (16.39%)
**Variants with >5% bias Single-end genome-based reads mapped with BWA**	15,473 (8.45%)	6,055 (44.83%)	21,528 (10.95%)
**Variants with >5% bias Single-end genome-based reads mapped with GEM**	17,794 (9.71%)	5,984 (44.30%)	23,778 (12.09%)
**Variants with >5% bias Single-end transcriptome-based reads mapped with GEM**	17,460 (9.53%)	5767 (42.69%)	23227 (11.80%)
**Variants with >5% bias Paired-end genome-based reads mapped with BWA**	9,449 (5.15%)	5812 (43.03%)	15261 (7.75%)

Numbers of variants sites (SNPs and indels) that show >0% or >5% difference in the mapping ratio of the reference and nonreference allele in simulations.

Table S3. Effect of filtering on mapped reads and expression quantification.

	Non-filtered	Filtered	% Difference
**Total reads**	7,180,867,302	7,042,040,776	1.93
**Mapped reads**	6,190,408,859	6,061,583,461	2.08
**Properly paired reads**	5,061,795,032	4,952,452,230	2.16
**Total quantified exons** (>0 reads in >0 individuals)	198,490	195,998	1.25
**Quantified exons** (>0 reads in >90% of individuals)	78,595	78,281	0.39
**Quantified autosomal exons** (>0 reads in >90% of individuals)	76,158	75,853	0.40
**Quantified genes** (>0 reads in >90% of individuals)	12,693	12,659	0.27
**Quantified autosomal genes** (>0 reads in >90% of individuals)	12,265	12,233	0.26
**Quantified autosomal exons tested for eQTL** (>0 reads in >90% of individuals (SNPs +/- 1 Mb from TSS)	76,155	75,851	0.40
**Quantified genes tested for eQTL**	12,263	12,232	0.25

Number of reads and quantified exons and genes before and after filtering biased reads.

Table S4. Overlap of eQTL datasets (Non-filtered and filtered) in the exon level.

		**Non-filtered data**		
		**eQTL exons**	**No eQTL exons**	**Total**
**Filtered data**	**eQTL exons**	5853	129	5982
	**No eQTL exons**	285	69656	69941
	**Total**	6138	69785	75923

Number of exons with and without significant eQTLs before and after filtering.

Table S5. Numbers and percentages of variants within the best eQTL exons in the lost, gained and common eQTL genes

	**Lost eQTL genes by filtering**	**Gained eQTL genes by filtering**	**Common eQTL genes**
**Exons with only SNPs**	69/119<br>57.98%	23/70<br>32.86%	1470/3253<br>45.19%

**Exons with only indels**	3/119<br>2.52%	3/70<br>4.29%	24/3253<br>0.74%
**Exons with at least 1 SNP and 1 indel**	24/119<br>20.17%	15/70<br>21.43%	687/3253<br>21.12%
**Exons with no variants**	23/119<br>19.33%	29/70<br>41.43%	1072/3253<br>32.95%

Of the lost eQTL genes that have no variants within the exons, 8/23 (34.79%) have variants either upstream or downstream of the exons that lead to filtering of reads. For the remaining 15/23 (65.21%), the filtering of reads in general leads to different normalization values, and the loss of the eQTL association.

Table S6. Number of exons with and without variants in common, lost and gained eQTL genes.

	**Common eQTL genes**	**Lost eQTL genes by filtering**	**Gained eQTL genes**
**Exons with variants**	2181	96	41
**Exons without variants**	1072	23	29
**Total**	3253	119	70

Lost eQTL exons are enriched with variants compared both to the common and gained eQTL exons (Fisher's exact test p-values<0.001).

Table S7. Genes that were highly significant before filtering biased reads with a difference in the $-\log_{10}$ of p-value before and after filtering >20 .

**Ensembl Gene ID**	**HGNC symbol**	**-log$_{10}$ non-filtered p-value**	**-log$_{10}$ filtered p-value**
ENSG00000233927	RPS28	51.3755	0.0508
ENSG00000105835	NAMPT	43.4829	0.0076
ENSG00000205581	HMGN1	38.4079	1.0855
ENSG00000198502	HLA-DRB5	30.2638	0
ENSG00000013573	DDX11	29.2523	0.9434
ENSG00000240563	L1TD1	28.7941	0
ENSG00000214401	KANSL1-AS1	27.5262	0
ENSG00000168028	RPSA	26.5622	0.2416

ENSG00000198754	OXCT2	24.9827	0
ENSG00000254772	EEF1G	23.5243	0.6853
ENSG00000037042	TUBG2	22.7877	0.3459

Figure S1

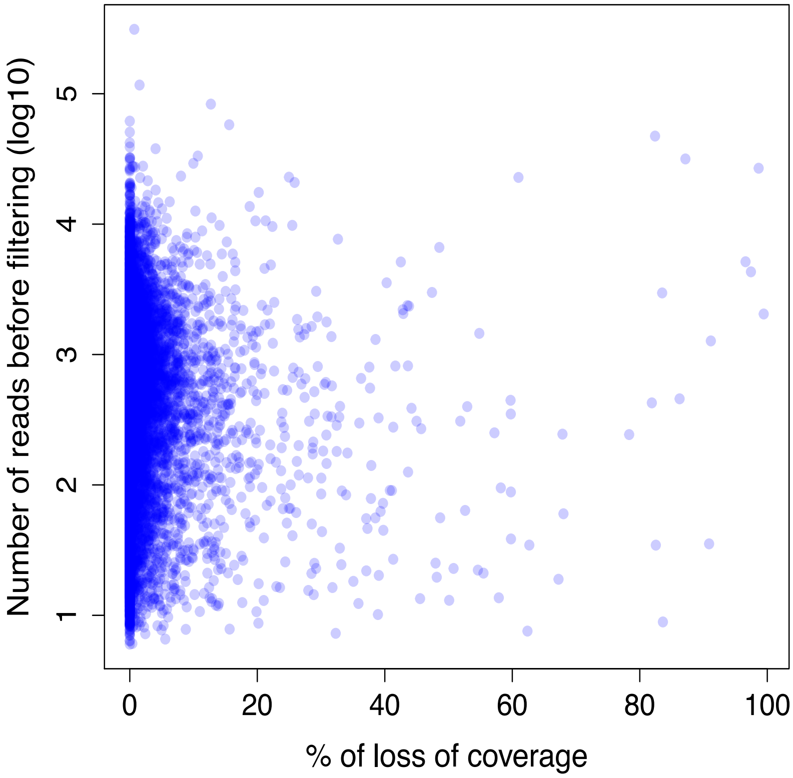

Figure S1. Proportional loss of coverage in filtering biased reads for each gene, compared to the log10 of the number of reads per gene in the original data.

Figure S2

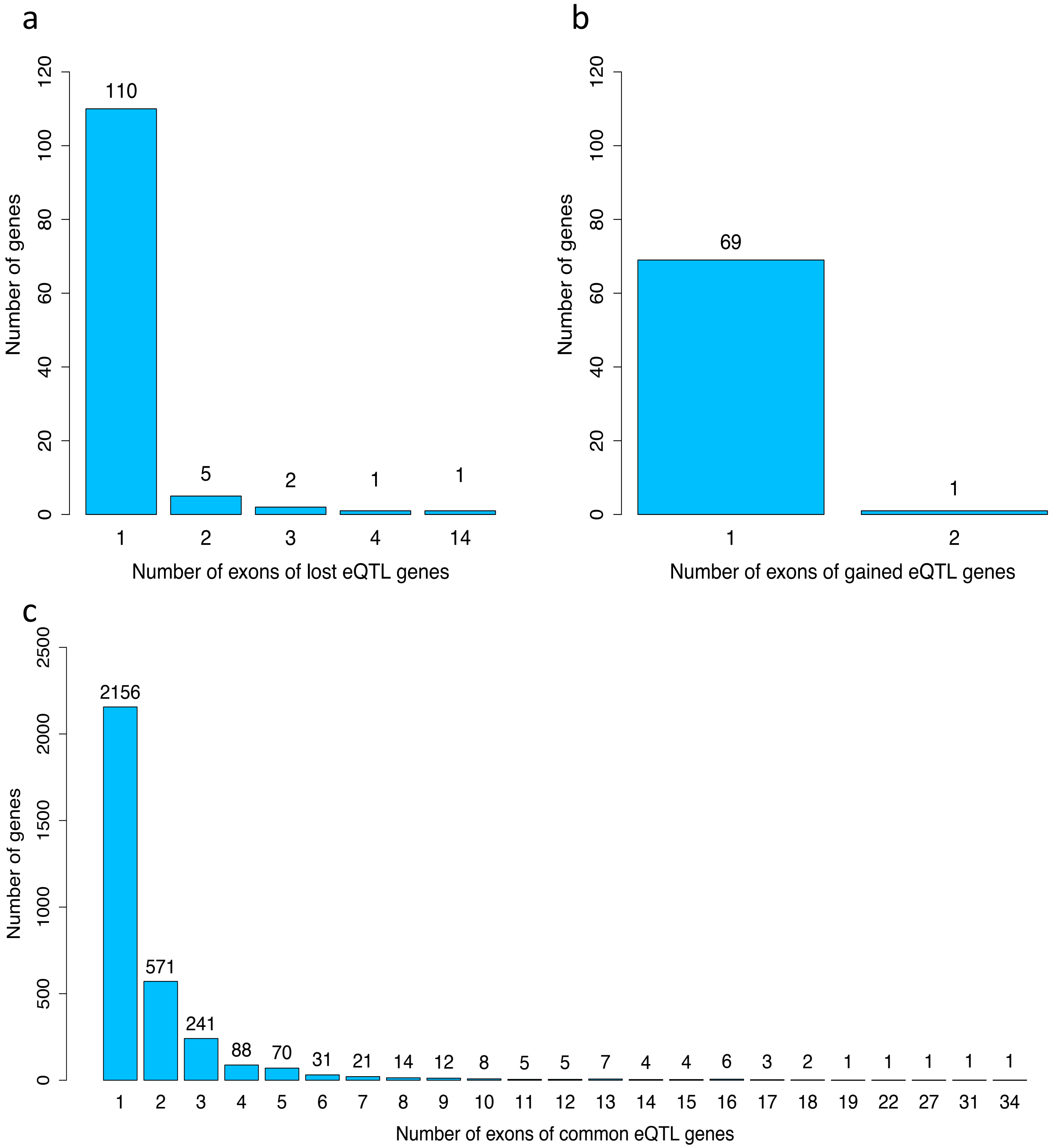

Figure S2. Distribution of the number of exons that passed the p-value threshold (significant exons) in eQTL genes in the non-filtered eQTL dataset for a) lost eQTL genes, b) gained eQTL genes, and c) common eQTL genes.

Figure S3

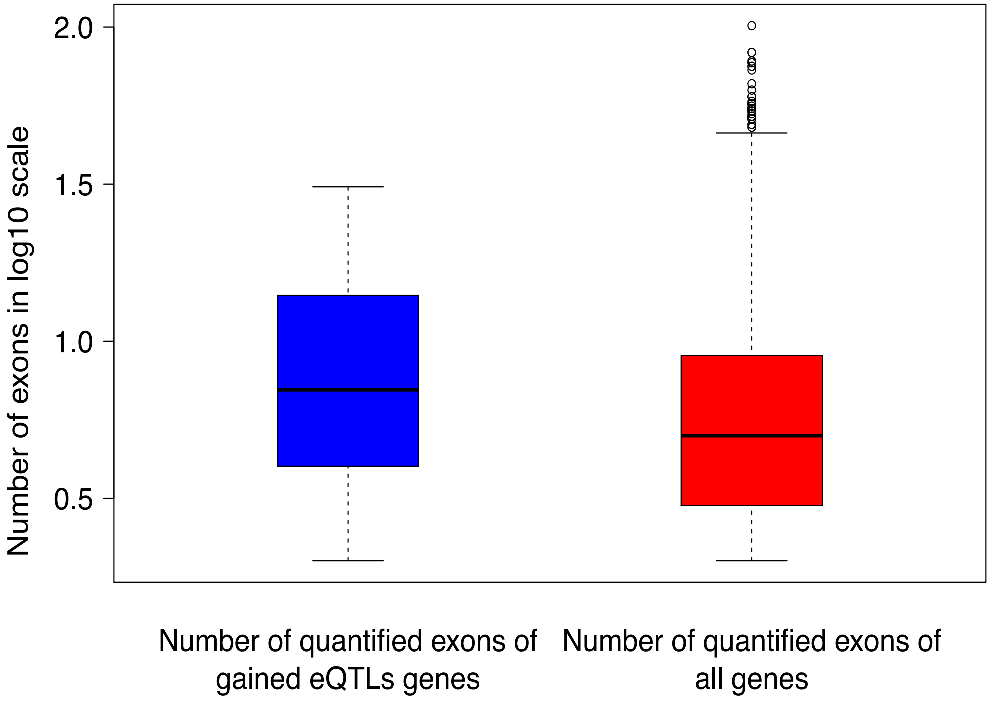

Figure S3. Distribution of the number of quantified exons in gained eQTL genes and all quantified genes (Mann-Whitney p-value<0.004). The higher the number of exons, the easier it is for a gene to pass the p-value threshold and be characterized as eQTL gene.
